# Trogocytosis of MHC-I/Peptide Complexes Derived from Tumors and Infected Cells Enhances Dendritic Cell Cross-Priming and Promotes Adaptive T Cell Responses

**DOI:** 10.1371/journal.pone.0003097

**Published:** 2008-08-29

**Authors:** Qian-Jin Zhang, Xiao-Lin Li, David Wang, Xiao-Cong Huang, J. Michael Mathis, Wei-Ming Duan, David Knight, Runhua Shi, Jonathan Glass, Dong-Qing Zhang, Lea Eisenbach, Wilfred A. Jefferies

**Affiliations:** 1 Department of Cellular Biology and Anatomy, Gene Therapy Program, Feist-Weiller Cancer Center, Louisiana State University Health Sciences Center, Shreveport, Louisiana, United States of America; 2 Medical School of Shanghai Jiao Tong University, Shanghai, China; 3 Department of Immunology, Weizmann Institute of Science, Rehovot, Israel; 4 The Michael Smith Laboratories and the Biomedical Research Centre, Departments of Medical Genetics, Microbiology and Immunology and Zoology, University of British Columbia, Vancouver, British Columbia, Canada; University of California Los Angeles, United States of America

## Abstract

The transporter associated with antigen processing (TAP) and the major histocompatibility complex class I (MHC-I), two important components of the MHC-I antigen presentation pathway, are often deficient in tumor cells. The restoration of their expression has been shown to restore the antigenicity and immunogenicity of tumor cells. However, it is unclear whether TAP and MHC-I expression in tumor cells can affect the induction phase of the T cell response. To address this issue, we expressed viral antigens in tumors that are either deficient or proficient in TAP and MHC-I expression. The relative efficiency of direct immunization or immunization through cross-presentation in promoting adaptive T cell responses was compared. The results demonstrated that stimulation of animals with TAP and MHC-I proficient tumor cells generated antigen specific T cells with greater killing activities than those of TAP and MHC-I deficient tumor cells. This discrepancy was traced to differences in the ability of dendritic cells (DCs) to access and sample different antigen reservoirs in TAP and MHC-I proficient versus deficient cells and thereby stimulate adaptive immune responses through the process of cross-presentation. In addition, our data suggest that the increased activity of T cells is caused by the enhanced DC uptake and utilization of MHC-I/peptide complexes from the proficient cells as an additional source of processed antigen. Furthermore, we demonstrate that immune-escape and metastasis are promoted in the absence of this DC ‘arming’ mechanism. Physiologically, this novel form of DC antigen sampling resembles trogocytosis, and acts to enhance T cell priming and increase the efficacy of adaptive immune responses against tumors and infectious pathogens.

## Introduction

Adaptive T cell immune responses play a critical role in controlling and destroying tumor cells. However, tumors that are deficient in components of the MHC-I antigen presentation pathway, such as TAP and MHC-I, are often observed to escape T cell responses [1]–[5]. This is due to failure in presentation of tumor antigens on the cell surface when TAP and MHC-I are absent [2]–[5]. TAP functions to transport cytosolic-generated peptides into the lumen of the endoplasmic reticulum (ER) for MHC-I binding, followed by transport of MHC-I/peptide complexes to the cell surface for T cell recognition.

Restoration of TAP and MHC-I expression in tumor cells has been demonstrated to increase T cell-based tumor antigen-specific immune responses [6]–[11]. Such responses consist of two phases, the induction and effector phases. The induction phase occurs in the early stages of the T cell immunity, initiating tumor-antigen recognition and antigen specific T cell generation and proliferation, while the effector phase occurs in later stages of the immune response, affecting T cell activation, recognition and destruction of antigen-expressing tumor cells. Using existing antigen specific T cells, many reports have confirmed that increased TAP and MHC-I expression in tumor cells restores the capacity for antigen presentation and thus enhances recognition and destruction of tumor cells by antigen specific T cells [10], [12]–[14]. These reports provide evidence that TAP and MHC-I expression facilitates T cell immunity in the effector phase. In addition to these, there are several studies indicating that restoration of TAP and MHC-I expression in tumor cells can augment T cell-based anti-tumor immune responses in both the induction and effector phases, by augmenting tumor immunogenicity. In these studies, tumor immunogenicity augmented by TAP and/or MHC-I expression was measured using tumor-cell-induced T cell responses to monitor the capacity for antigen processing and presentation of the tumor [10], [14] or through the use of tumor-bearing mice as a model to determine the length of survival as a quotient for adaptive immune responses *in vivo* [10], [13]. Since these studies did not distinguish between the induction versus the effector phases of an adaptive immune response, it is still uncertain if enhanced tumor immunogenicity is due to an increase in activity of tumor antigen-specific T cells during the induction phase of the T cell response.

Induction of anti-viral and anti-tumor T cell-based immunity requires professional antigen presenting cells, such as bone-marrow derived DCs (BM-DCs) [15]–[18]. DCs acquire antigen proteins from apoptotic or necrotic tumor cells and process and present them via the MHC-I antigen presentation pathway to the surface for T cell priming. This processing pathway is termed cross-presentation and is an important mechanism for T cell generation [16], [19]. A study on allorecognition has provided additional evidence for the effectiveness of cross-presentation by showing that DCs were capable of acquiring substantial levels of allogeneic MHC-I from donor cells [20]. In this process, DCs appear to capture MHC-I and likely present antigens directly to T cells. The physiological relevance of this mechanism in vivo has not been established and it is unclear whether such a mechanism is suitable for the induction of anti-viral or anti-tumor T cell responses in addition to conventional DC cross-presentation.

In the present study, we examined different forms of a model antigen VSV-Np derived from the vesicular stomatitis virus nucleocapsid protein to address the induction phase of the T cell response and determined whether tumor cells that have reconstituted TAP and MHC-I expression can prime antigen specific T cells with increased killing via DC-sampling of surface MHC-I/peptide complexes.

## Results

### TAP and MHC-I expression in tumors increases tumor-antigen specific immunity both *in vitro* and *in vivo*

**Figure 1:**
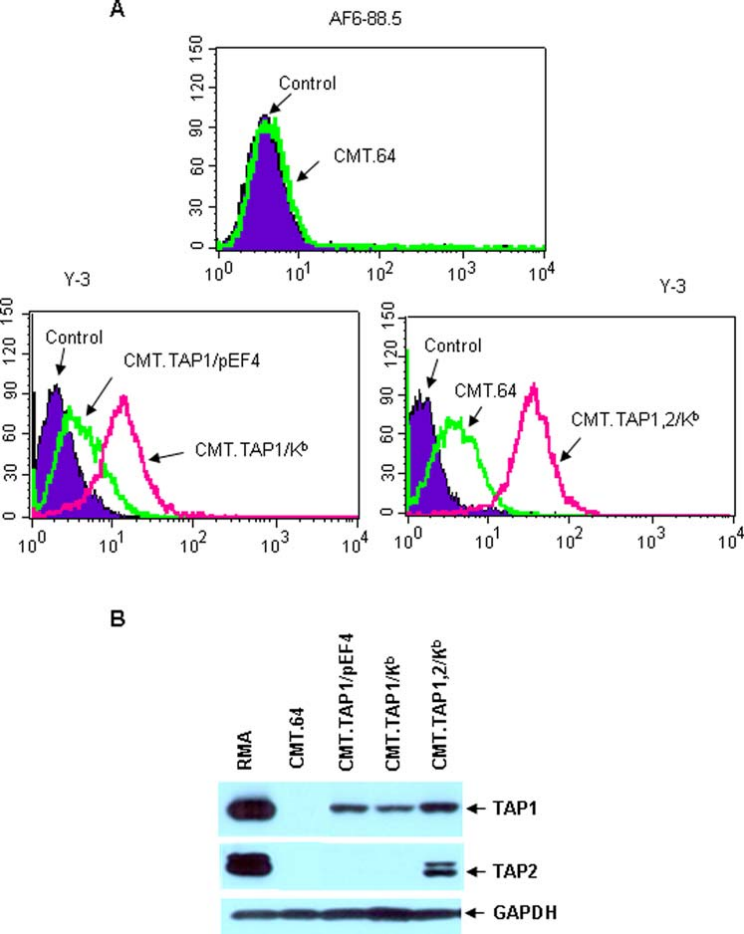
Expression of transfected TAP and K^b^ molecules in CMT.64 cells. K^b^ and TAP expression in CMT.64 transfectants was examined. A) FACS assay was performed to detect expression of the K^b^ molecule by direct immunostaining using R-PE-conjugated K^b^-specific mAb AF6-88.5, and R-PE-conjugated K^k^-specific Ab 36-7-5 was used as a control (top panel). In addition, indirect immunostaining was performed using Y-3 Ab against K^b^ followed by labeling with FITC-conjugated goat anti-mouse IgG Ab. The samples labeled with only FITC-conjugated goat anti-mouse IgG Ab were used as controls (meddle panel). B) Western blots were performed to detect mouse TAP1 and TAP2 expression using the respective polyclonal antibodies respectively (see Material and Methods). GAPDH protein was detected in each sample as the loading control.

**Figure 2:**
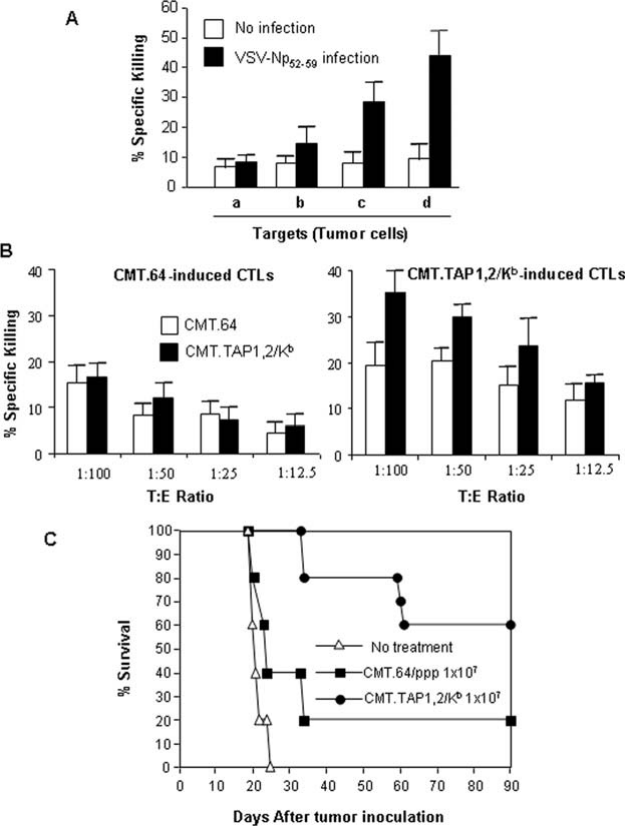
CMT.64 TAP/MHC I transfectants but not wild-type CMT.64 cells present antigens and induce an immune response. *In vitro* cytotoxicity assays and *in vivo* challenge assays were performed to determine the antigen presentation by the tumor cells and anti-tumor immune response. A) Determination of antigen presentation: Cells were infected with VV-VSV-Np_52-59_ at 1∶20 (m.o.i) overnight, labeled with ^51^Cr and used as the targets. The VSV-Np_52–59_ specific splenocyte-derived CTLs were generated by immunization of mice with VV-VSV-Np_52–59_ at 2×10^7^ (pfu) viruses per mouse. The target to effector ratio used was 1∶100. a – CMT.64; b – CMT.TAP1/pEF4; c – CMT.TAP1/K^b^ and d – CMT.TAP1,2/K^b^. The mean value of the results from two experiments is shown. B) Determination of anti-tumor immune response: CMT.64 and CMT.TAP1,2/K^b^ immunized splenocytes were used as two effectors and ^51^Cr-labeled CMT.64 and CMT.TAP1,2/K^b^ cells were used as targets. The mean value of the results from two experiments is shown. C) Left: Mice were immunized ip. with 1×10^7^ γ-irradiated CMT.TAP1,2/K^b^, CMT.64/ppp cells (experimental control) or PBS (negative control. After day 20 of immunization, the mouse was challenged ip. with 2.5×10^5^ cells/mouse CMT.TAP1,2/K^b^ cells. Each group contained 10 mice. Time of morbidity was recorded. Statistical analysis of survival curves is shown below: P>0.05 was used for comparison between CMT.64/ppp-immunized and negative control groups; P<0.001 was for the comparison between CMT.TAP1,2/K^b^-immunized and negative control; P<0.05 was used for comparison between the CMT.TAP1,2/K^b^- and CMT.64/ppp-immunized groups. It should be noted that CMT.64/ppp, instead of CMT.64, were used to immunize mice is to control for immune responses against components of the three vectors used to transfect TAP and K^b^.

To evaluate TAP and MHC-I function in the induction phase of the T cell response, we transfected TAP1, TAP2 and/or K^b^ genes into TAP and MHC-I deficient CMT.64 cells. All transfectants expressed the relevant gene(s) (Fig. 1A and 1B). K^b^ expression in CMT.64 cells was not detected by a conformational dependent AF6-88.5 mAb but was visible using Y-3 mAb, and the level of expression in the cells was lower than the level shown in CMT.1,2/K^b^ cells. CMT.TAP1/pEF4 cells expressed a level of K^b^ lower than CMT.TAP1/K^b^ (Fig. 1A). All gene-transfectants expressed mouse TAP1 while only CMT.TAP1,2/K^b^ expressed TAP2 (Fig. 1B). The empty vector-transfectant CMT.64/ppp had K^b^ and TAP expression levels similar to CMT.64 (data not shown). Functional assays indicated that presentation of a K^b^-restricted epitope VSV-Np_52–59_ derived from VSV-Np protein varied among these transfectants (Fig. 2A). Since this epitope can be transported onto the surface of TAP-proficient and TAP1-expressing cells but not TAP-negative cells [2], our results indicated that presentation of this epitope correlated with levels of K^b^ molecules as well as with TAP expression.

Next, we performed *in vitro* and *in vivo* experiments to determine whether the TAP and MHC-I proficient CMT.TAP1,2/K^b^ was able to induce tumor-antigen specific immunity. For *in vitro* experiments, γ-irradiated CMT.64 and CMT.TAP1,2/K^b^ were used to immunize mice and the two immunized splenocyte populations were compared for tumor-antigen recognition using a ^51^Cr-release assay. CMT.64-generated splenocytes recognized CMT.TAP1,2/K^b^ at low levels similar to CMT.64 (figure 2B left-panel), while the CMT.TAP1,2/K^b^-generated splenocytes killed CMT.TAP1,2/K^b^ with higher efficiency than CMT.64 (Fig. 2B right-panel). This was also observed in an *in vivo* challenge experiment. The CMT.TAP1,2/K^b^-immunized mice challenged with live CMT.TAP1,2/K^b^ had a longer survival rate than the CMT.64/ppp-immunized mice (P<0.05, Figure 2C). Although survival of the CMT.64/ppp-immunized group tended to be longer than the negative control group (non-immunized mice), statistical analysis showed no significance (P>0.05). In contrast to immunocompetent mice, protection by immunization with CMT.TAP1,2/K^b^ cells was not observed in a nude mouse model that lacks antigen specific T cells (data not shown). These experiments demonstrated that expression of surface MHC-I/peptide complexes increased protection in tumor-bearing mice, likely due to increased tumor-antigen specific immunity.

In spite of TAP and MHC-I expression increasing tumor-antigen specific immunity, the importance of the induction phase of this response was still not addressed because clonal selection in response to the CMT.TAP1,2/K^b^ and the CMT.64/ppp cells may be dependent on factors other than TAP and K^b^ expression. For example, different levels of antigen protein expression may have influenced induction of the immune response. To resolve this question and determine if TAP and K^b^ expression can potentiate the induction of tumor-antigen specific immunity, a model antigen was introduced into the tumor cells to induce production of antigen-specific T cells and to determine T cell activities.

### Efficient killing activity of antigen-specific CTLs generated by γ-irradiated TAP and K^b^ expressing cells infected with VV carrying mini-genes

**Figure 3:**
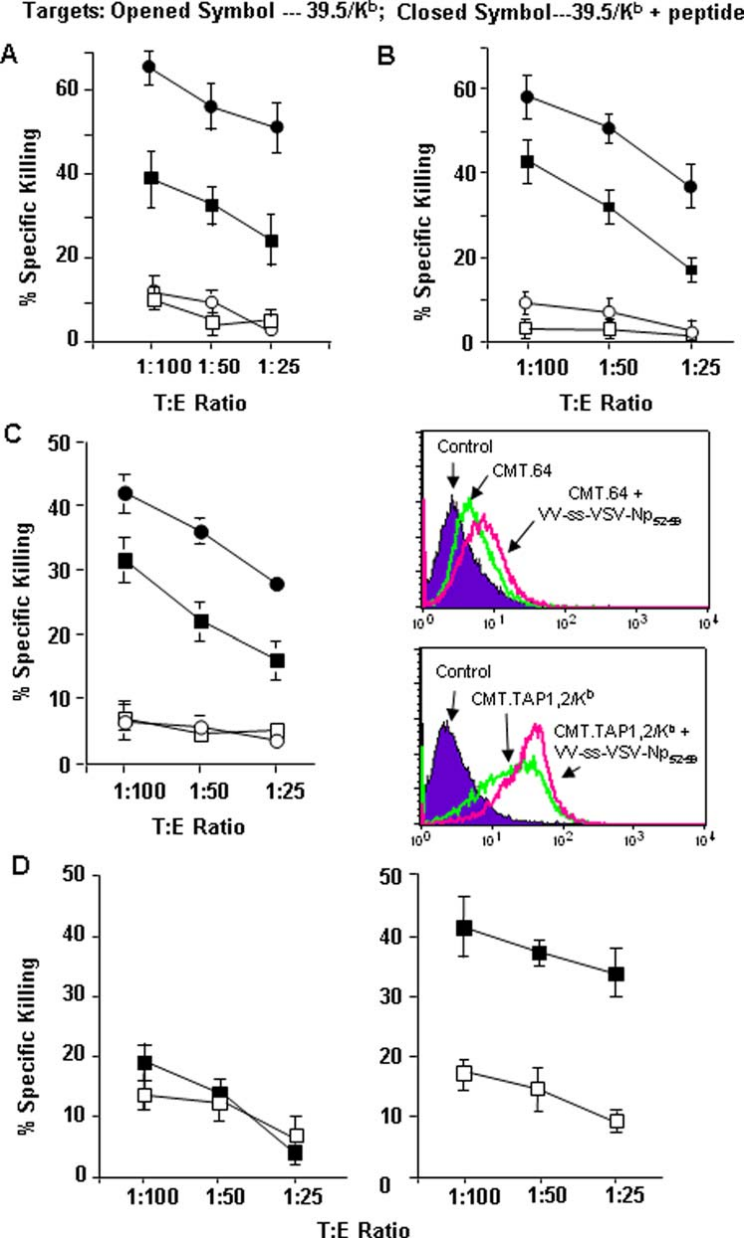
Increased killing activities of VSV-Np_52–59_ specific CTLs generated by TAP and K^b^ expressing tumor cells. Standard 4-h ^51^Cr-release assays were performed to detect the killing activities of the spleen-derived antigen specific CTLs. The CTLs were generated by immunization of 1×10^7^ cells/mouse γ-irradiated CMT.64, CMT.TAP1/pEF4, CMT.TAP1/K^b^ or CMT.TAP1,2/K^b^ cells that were infected with VV-VSV-Np_52–59_ B or VV-ss-VSV-Np_52–59_ virus at 1∶20 (m.o.i) overnight and re-stimulated with VSV-Np_52–59_ peptide *in vitro*. The ^51^Cr-labeled 39.5/K^b^ cells pulsed with or without VSV-Np_52–59_ peptide were used as targets. A) Square symbol — CTLs generated by CMT.64+VV-VSV- Np_52–59_; Round symbol — CTLs generated by CMT.TAP1/K^b^+VV-VSV-Np_52–59_. The mean value of the results from three experiments is shown. B) Square symbol — CTLs generated by CMT.TAP1/pEF4+VV-VSV-Np_52–59_; Round symbol — CTLs generated by CMT.TAP1/K^b^+VV-VSV-Np_52–59_. The mean value of the results from three experiments is shown. C) Left: Square symbol — CTLs generated by CMT.64+VV-ss-VSV-Np_52–59_; Round symbol — CTLs generated by CMT.TAP1,2/K^b^+VV-ss-VSV-Np_52–59_. The results represented the mean value from three experiments. Right: FACS assay was performed to detect surface K^b^ expression. D). CTLs were generated by immunization of mice with 1×10^7^ pfu γ-irradiated (Left panel) and non-irradiated (Right panel) VV-VSV-Np_52–59_. The results represent the mean value from two experiments.

To evaluate the effects of TAP and K^b^ expression in tumor cells on enhancement of T cell priming, we performed a ^51^Cr-release assay to determine antigen-specific T cell activity. VV-VSV-Np_52–59_-infected CMT.TAP1,2/K^b^ and CMT.64 were inoculated i.p. into naïve mice and the immunized splenocytes were used as a source of T cells. Since the VV carried a VSV-Np_52–59_ minigene, the viral infected cells generated only the peptide epitope but not the entire VSV-Np protein. It is important to note that, while both cell lines expressed equivalent amounts of viral antigen, the CMT.TAP1,2/K^b^ cells could present VSV-Np_52–59_ peptide on their cell surface whereas the CMT.64 cells could not (Fig. 2A). As shown in Fig. 3A, the CMT.TAP1,2/K^b^-generated CTLs had killing activity higher than the CMT.64-generated CTLs.

We subsequently compared cytolytic activities of two T cell populations generated by immunization of VV-VSV-Np_52–59_-infected CMT.TAP1/pEF4 or CMT.TAP1/K^b^ cells. These two cell lines expressed TAP1 only and therefore the transport of the VSV-Np_52–59_ peptide into the lumen of the ER and subsequent expression of MHC I/peptide complexes at the cell surface may be reduced in comparison with TAP1 and 2 expressing cells (see Fig. 2A). In this setting the capacity for T cell priming between these two cell lines differs in the levels of MHC I and MHC-I/ peptide complexes displayed at the cell surface. With higher K^b^ expression, the CMT.TAP1/K^b^ cells presented more VSV-Np_52–59_ peptide on the cell surface than the CMT.TAP1/pEF4 cells when both cell lines were infected with VV-VSV-Np_52–59,_ as demonstrated by CTL killing (Fig. 2A). The CMT.TAP1/K^b^-generated splenocytes exhibited a higher killing activity than the CMT.TAP1/pEF4-generated splenocytes (Fig. 3B). Furthermore, we designed an additional experiment to address this issue through direct infection of CMT.64 and CMT.TAP1,2/K^b^ cells with a VV-ss-VSV-Np_52–59_ virus. Peptide epitopes governed by an ER-signal sequence (ss) can bypass the requirement of TAP function to be transported into the ER lumen where the peptides are cleaved to remove the signal sequence for MHC-I binding [21]–[23]. In this experimental system, synthesized peptide epitopes can freely enter the ER lumen and thus the key component for antigen presentation appears to be the expression of K^b^ molecules within the secretory compartment. In this experiment, the immunization of mice with VV-ss-VSV-Np_52–59_-infected CMT.TAP1,2/K^b^ cells generated a CTL population with killing activity higher than those immunized with the viral infected CMT.64 cells (Figure 3C left panel). The viral infection slightly increased the surface K^b^ expression in both CMT.64 and CMT.1,2/K^b^ cells (Fig. 3 C right panel). Thus, these results suggest that MHC-I expression contributes to the greater efficiency of T cell priming of K^b^-expressing CMT.64 transfectants.

To confirm that the VV-VSV-Np_52–59_ viruses themselves did not prime antigen specific T cell generation, we compared the abilities of the irradiated and non-irradiated VV-VSV-Np_52–59_ viruses to induce VSV-Np_52–59_ epitope specific T cells. Two splenocyte populations generated from the mice inoculated with γ-irradiated and non-irradiated VV-VSV-Np_52–59_ were compared with respect to their killing activities. CTLs generated by live, but not irradiated VV-VSV-Np_52–59_ showed killing activity (Fig. 3D). These results indicate that irradiation abrogated CTL generation.

### Low dose dependency of VV-VSV-Np-infected CMT.TAP1,2/K^b^ cells in priming a VSV-Np_52–59_-specific CTL population

**Figure 4:**
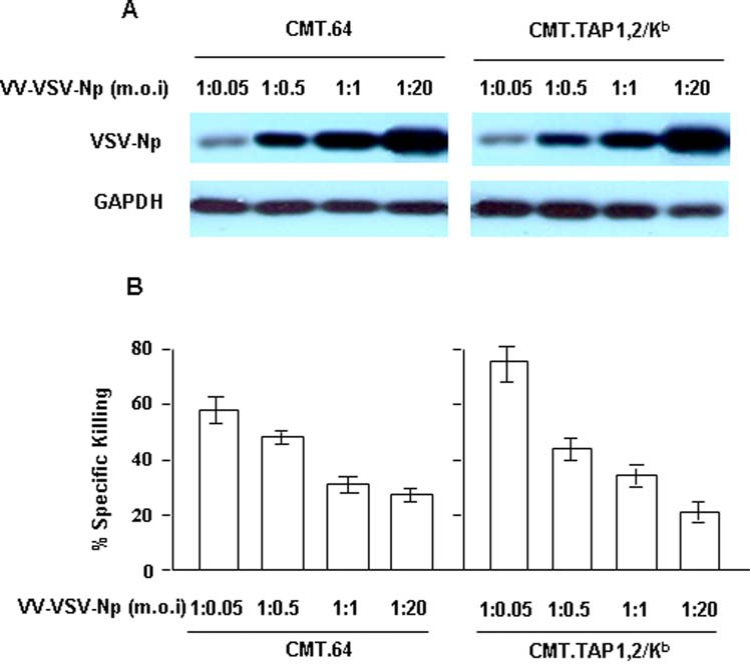
Increased killing activity of CTLs generated by CMT.TAP1,2/K^b^ infected with the lowest dose of VV-VSV-Np. The CMT.TAP1,2/K^b^ and CMT.64 cells were infected with VV-VSV-Np virus at varied m.o.i. A) Western blots were performed to detect VSV-Np protein expression. B) Standard 4-h ^51^Cr-release assays were conducted to determine the activities of the CTLs generated by varied viral-dose infected CMT.TAP1,2/K^b^ and CMT.64 cells (1×10^7^ cells/mouse). The ^51^Cr-labeled 39.5/K^b^ cells pulsed with VSV-Np_52–59_ peptide were used as targets. 1∶50 target to effector ratio is shown. The mean value of the results from two experiments is shown.

To test the effect of TAP and K^b^ on T cell priming by antigens from the entire VSV-Np protein, we infected CMT.TAP1,2/K^b^ and CMT.64 cells with 1∶20 pfu VV-VSV-Np that contained the full-length gene. Cytolytic activities of two splenocyte populations from the immunized mice were tested. Unexpectedly, killing activities of the two CTL populations were equally potent (data not shown), suggesting that the MHC-I/peptide complexes did not participate in T cell priming under these experimental conditions. One possible explanation for this observation is that cells infected with VV-VSV-Np at 1∶20 (m.o.i) generated amounts of the VSV-Np protein sufficient for T cell priming, thereby obscuring the effects of surface antigen-loaded K^b^ complex on the induction of T cells. In order to understand this phenomenon, the CMT.TAP1,2/K^b^ and CMT.64 cells were infected at a reduced m.o.i of the VV-VSV-Np, and VSV-Np protein expression and T cell activities of the viral infected cell-generated CTLs were examined by Western blot and ^51^Cr-release assay respectively (Fig. 4). At a dose of 1∶20 (m.o.i) infection, both cell lines expressed a large but equal amount of the VSV-Np protein, which decreased with reduced doses of viral infection. However, at the minimum of 1∶0.05 (m.o.i), VSV-Np protein was still detectable (Fig. 4A). When assayed for CTL activity, the CTL population generated by viral infected CMT.TAP1,2/K^b^ cells at the lowest dose showed a killing activity significantly greater than the CTL population generated at the equivalent m.o.i using viral-infected CMT.64 cells (Fig. 4B). However, the increased activities were not observed in CTL populations generated by the viral infected CMT.TAP1,2/K^b^ cells at doses higher than 1∶0.05 (m.o.i). Our results demonstrate that: 1) low but not high protein antigen expression primed a T cell population with increased killing activity in CMT.TAP1,2/K^b^ cells; and 2) T cell cytolytic activities were decreased when antigen protein expression was increased.

### Frequency of antigen specific T cells is not increased in animals immunized with viral infected CMT.TAP1/K^b^ and CMT.TAP1,2/K^b^ cells

**Figure 5:**
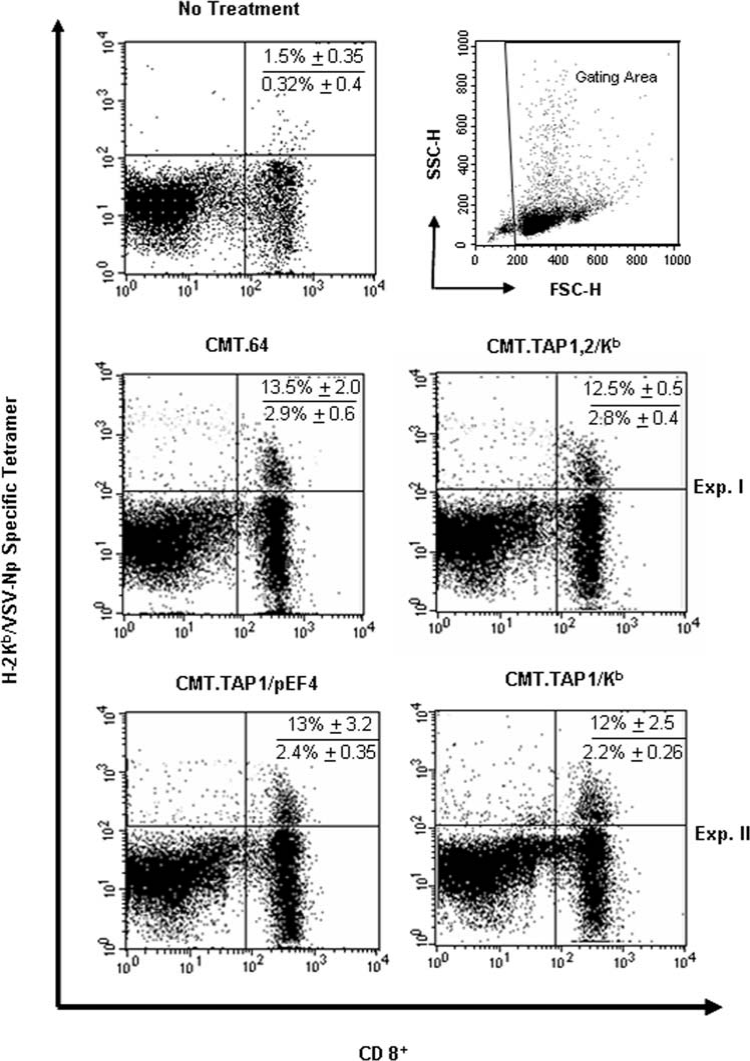
Similar frequencies of VSV-Np_52–59_ specific T cells generated by immunization of different tumor cells. Tumor cells were infected with VV-VSV-Np_52–59_ overnight and were γ-irradiated. The cells were then injected into mice. After immunization, the frequency of VSV-Np_52–59_ specific CD8^+^ T cells derived from splenocytes was analyzed by FACS assay using double staining with an anti-mouse CD8a antibody and a K^b^/VSV-Np_52–59_ specific tetramer. The gating area was set up to include all cells except erythrocytes and debris (right plot at top panel). Exp. I and Exp. II indicate that tetramer assays were performed separately. One out of two tetramer assays is shown. The numbers inserted in each picture represent the mean value of results (mean±SEM) from two experiments. Numbers shown in top are percentages of tetramer positive cells in total CD8^+^ T cells. Numbers shown in bottom are percentages of tetramer positive cells in total splenocytes.

To determine if the number of antigen specific T cells influences their killing activities, a K^b^/VSV-Np_52–59_ tetramer assay was performed in two pairs of splenocyte-populations from immunized mice to quantify the percentage of antigen specific CD8^+^ cytolytic T cells. VV-VSV-Np_52–59_ infected CMT.64 and CMT.TAP1,2/K^b^ generated splenocyte-populations were compared and viral infected CMT.TAP1/pEF4 and CMT.TAP1/K^b^ generated splenocyte-populations were used in another comparison. All immunized splenocytes were stimulated *in vitro* with VSV-Np_52–59_ peptide for 5 days and then each was divided into two aliquots; one was used as effectors in cytotoxicity assays as shown in Fig. 3A and B and another was used to quantifyVSV-Np_52–59_ specific and K^b^–restricted T cells in the tetramer assay (Fig. 5). Results showed that neither the percentages of tetramer positive cells in total splenocytes nor the total CD8^+^ T cells were increased in the CMT.TAP1,2/K^b^-generated population, compared with the CMT.64-generated population (Fig. 5, Exp. I). In addition, similar results were observed in the CMT.TAP1/K^b^-generated and CMT.TAP1/pEF4-generated populations (Fig. 5, Exp. II). These results together with the results shown in Fig. 3A and B suggest that TAP and K^b^-expressing cells generated T cell populations with different effector potency instead of increasing the number of antigen-specific T cells.

### Uptake of surface MHC-I/peptide complex from the tumor cells by BM-DCs

Since the observed increase in T cell response was based on priming by γ-irradiated cells, it was possible that DCs play an important role in antigen uptake from the apoptotic cells for T cell priming via a cross-presentation pathway. If so, the question remains as to why, if equal amounts of antigen expression in CMT.64 and CMT.TAP1,2/K^b^ (by viral infection) were taken up by DCs, do differences in cross-priming explain the differences in T cell responses. To answer this, we focused on examining the surface MHC-I/peptide complex of the tumor cells to determine if DCs are able to utilize them as an additional source of antigen for T cell priming.

**Figure 6:**
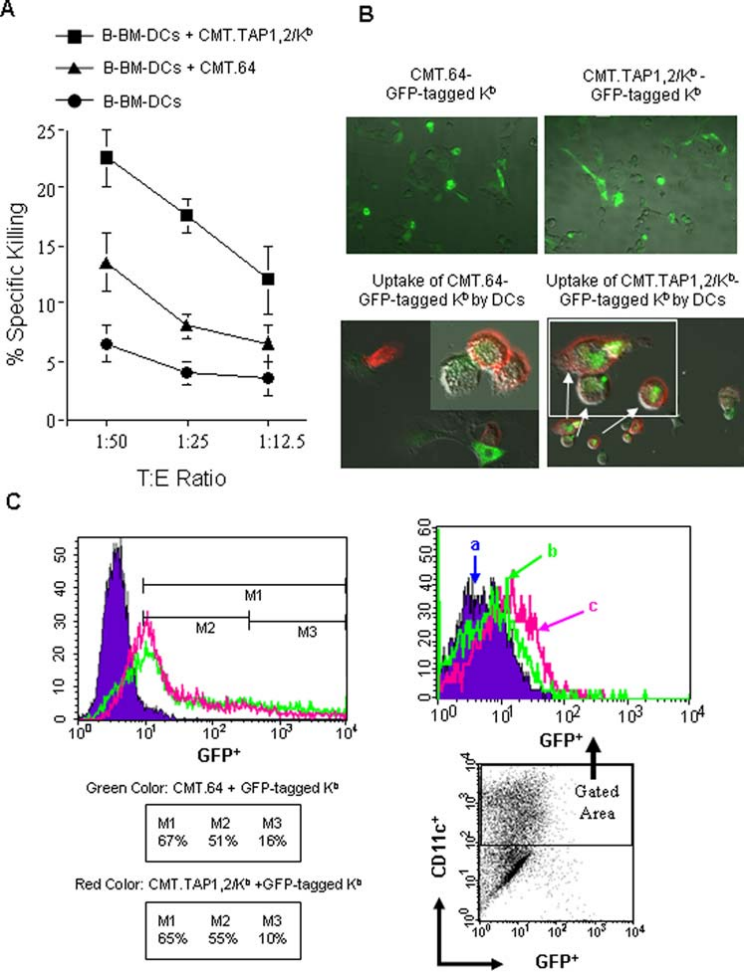
Uptake of the MHC-I/peptide complex and expression on the surface of the tumor cells by BM-DCs. Uptake of K^b^ molecules by BM-DCs was quantified using *in vitro* cytotoxicity assays, multi-channel fluorescence microscopy and FACS assays. A) B-BM-DCs were incubated with γ-irradiated CMT.64 or CMT.TAP1,2/K^b^ cells that were infected with 1∶10 pfu VV-VSV-Np_52–59_. The matured B-BM-DCs were used as ^51^Cr-labeled targets in cytotoxicity assays. The mean value of the results (Mean±SEM) from two experiments is shown. B). C-BM-DCs were co-cultured with γ-irradiated CMT.64 and CMT.TAP1,2/K^b^ cells transiently transfected with GFP-tagged K^b^ vector (green color). After 6 hours incubation, DCs were treated with LPS overnight. DC samples were then labeled with Alexa Fluor 647 anti-mouse CD11c antibody (red color), and dual-channel fluorescence was visualized by using a Zeiss AxioObserver Z1 widefield microscope. The top-panel indicates that tumor cells transiently expressed GFP-tagged K^b^ and that most cells were weakly GFP positive. The bottom-panel (including inserted images) indicates that DCs took up the green label from tumor cells. Arrows show the same cells enlarged. C). FACS assays was performed to detect GFP expression. Left panel: CMT.64 cells (green) and CMT.TAP1,2/K^b^ cells (red) transiently transfected with GFP-tagged K^b^ vector overnight and GFP expression was detected. CMT.64 cells without transfection (Blue) were used as control. CMT.TAP1,2/K^b^ cells without transfection has fluorescence intensity similar to CMT.64 cells (data not shown). In inserted table, M1 shows the percentage of total GFP positive cells; M2 shows the percentage of weak GFP positive cells and M3 shows the percentage of high GFP positive cells. Right panel: C-BM-DCs, that were prepared similar to those depicted in B, were labeled with PE-conjugated anti-mouse CD11c. GFP-fluorescence intensity of DCs in a histogram at top was gated for CD11c positive cells (shown in a dot-plot at bottom). a — DCs incubated with γ-irradiated CMT.64 cells; b — DCs incubated with γ-irradiated, GFP-tagged K^b^ transfected CMT.64 cells; c — DCs incubated with γ-irradiated, GFP-tagged K^b^ transfected CMT.TAP1,2/K^b^ cells.

BM-DCs were obtained from BALB/c mice (designated as B-BM-DCs) that express H-2^d^ (K^d^ and D^d^) molecules. If B-BM-DCs incubated with γ-irradiated CMT.TAP1,2/K^b^ infected with VV-VSV-Np_52–59_ could be recognized by the VSV-Np_52–59_ specific T cells, it would suggest that the K^b^/peptide complex from the donor cells were sampled, acquired and expressed on the plasma membrane of recipient DCs and this facilitated functional recognition of the transferred MHC-I/antigen complexes. We performed a ^51^Cr-release assay to detect donor K^b^/peptide complex presentation by B-BM-DCs. The CMT.64 cells were used as a control, as unlike the CMT.TAP1,2/K^b^, the VSV-Np_52–59_ epitope is not transported onto the cell surface in association with K^b^ molecules (Fig. 2A). VSV-Np_52–59_ CTLs killed the B-BM-DCs incubated with CMT.TAP1,2/K^b^ more efficiently than those incubated with CMT.64 (Figure 6A). In addition to monitoring cytolytic activity, the surface MHC-I transfer event was directly observed using a GFP-tagged-K^b^-vector system. CMT.TAP1,2/K^b^ and CMT.64 cells were transiently transfected with a GFP-tagged-K^b^-vector overnight and GFP expression was detected by fluorescence microscopy and FACS assay (Fig. 6B and C). After overnight transfection, GFP expression could be detected in approximately 65–67% of cells; however, within these cells, the GFP expression was weakly expressed in 51–55% of the transfected cells. A transient GFP expression system was necessary since the GFP vector required neomycin screening, which was already present in our current cell lines (see Material and Methods). The GFP-tagged-K^b^-transfectants were then γ-irradiated and incubated with C57BL/6-derived BM-DCs (designated as C-BM-DCs) to follow DC' uptake of GFP-K^b^ molecules. The GFP-tagged MHC-I uptake was determined by fluorescence microscopy and FACS analysis. Fluorescence microscopic results (Fig. 6B bottom) revealed little uptake of the GFP-tagged K^b^ by DCs from co-culture with CMT.64 cells while intense green color was visualized in DCs co-cultured with CMT.TAP1,2/K^b^ cells. These results suggested that DCs are able to directly acquire portions of plasma membrane from the tumor cells. FACS analysis demonstrates that the DC population co-cultured with GFP-tagged-K^b^-transfected CMT.TAP1,2/K^b^ cells acquires a fluorescence intensity higher than the DCs co-cultured with GFP-tagged-K^b^-transfected CMT.64 cells (Fig. 6C, right panel). These results indicate that DCs can efficiently acquire and re-utilize transferred surface MHC-I/Peptide complexes from TAP expressing tumor cells through a trogocytosis-like process [24].

### T cell priming by BM-DCs activated by acquiring H-2K^b^/VSV-Np complexes from the surface of infected cells

**Figure 7:**
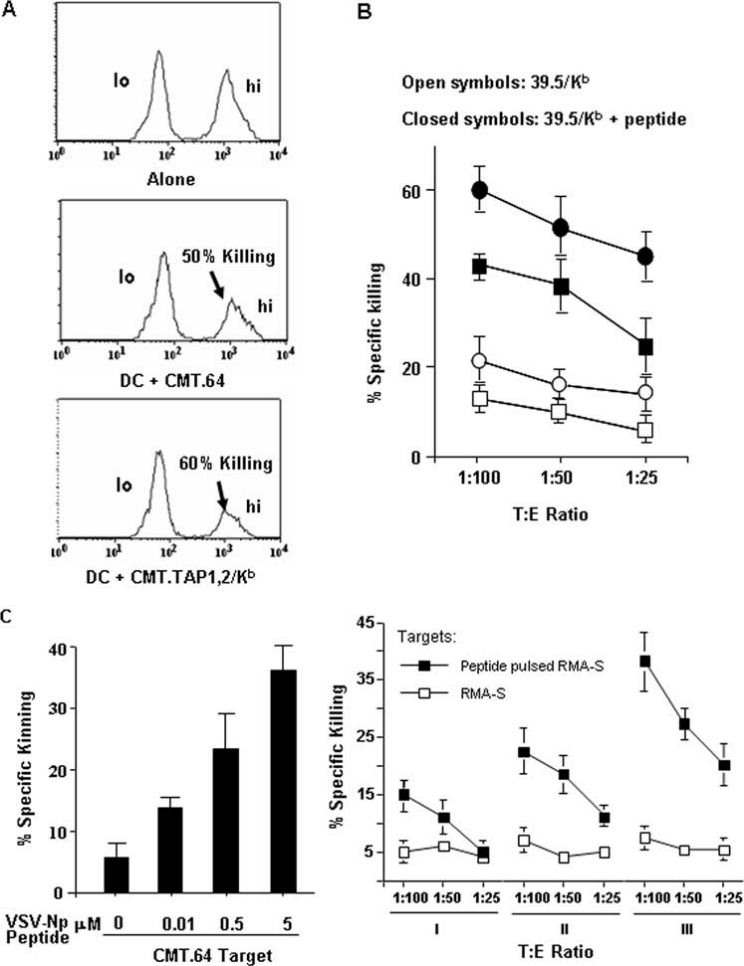
T cell priming by BM-DCs activated by H-2K^b^/VSV-Np complexes on the surface of tumor cells. A) B-BM-DCs were incubated with γ-irradiated CMT.64 or CMT.TAP1,2/K^b^ cells that were infected with 1∶10 pfu VV-VSV-Np_52–59_. After extensive washing, B-BM-DCs were immunized to naïve C57BL/6 mice to perform *in vivo* cytotoxicity assays. The C57BL/6 mice without immunization were used as controls (see material and methods for detail). One representative of two experiments is shown. In the two experiments, the mean value (percentage) of killing was 57.5%±2.5 (Mean % killing±SEM) for DCs co-cultured with γ-irradiated and VV-VSV-Np_52–59_-infected CMT.TAP1,2/K^b^ and 46.5%±3.5 (Mean % killing±SEM) for DCs co-cultured with γ-irradiated and VV-VSV-Np_52–59_-infected CMT.64. B) C-BM-DCs were treated as A) and immunized to naïve C57BL/6 mice. Spleens from the immunized mice were used as CTLs. Standard 4-h ^51^Cr-release assays were performed and the 39.5/K^b^ cells pulsed with or without VSV-Np_52–59_ were used as targets. The mean value of the results from three experiments is shown. C). Standard 4-h ^51^Cr-release assays were performed. Left panel: CMT.64 cells pulsed with 0.01, 0.5 and 5 µM VSV-Np_52–59_ peptide were used as target cells, and CTLs were generated by immunization with 39.5/K^b^ cells pulsed with VSV-Np_52–59_ peptide. Right panel: C-BM-DCs were incubated with γ-irradiated CMT.64 cells that were pulsed with 0.01, 0.5 or 5 µM VSV-Np_52–59_ peptide. After extensive washing, the DCs were injected into mice to generate VSV-Np52–59 epitope specific CTLs (splenocytes). RMA-S and VSV-Np52–59 (2 µM) peptide-pulsed RMA-S cells were used as targets. I, II and III indicated that CTLs generated by immunization of DCs co-cultured with CMT.64 cells pulsed with 0.01 (I), 0.5 (II) and 5 (III) µM peptide. The results represent the mean value of the results from two experiments.

An important issue to consider is if the amount of MHC-I/peptide complexes taken up by DCs, is it sufficient to induce production of antigen specific T cells. To address this issue, both B-BM-DCs and C-BM-DCs were injected into C57BL/6 mice to generate T cell responses. Differences exist in the MHC-I molecules expressed by the B-BM-DCs versus the C-BM-DCs that can participate in T cell priming. If the donor MHC-I-loaded B-BM-DCs can not efficiently induce T cell responses while the MHC-I-loaded C-BM-DCs can induce efficient T cell responses, this would explain the observation that antigens from exogenous MHC-I/peptide complexes alone are not sufficient to induce immune responses. To test this possibility, we performed *in vivo* cytotoxicity assays for B-BM-DCs. B-BM-DCs were loaded with either γ-irradiated, VV-VSV-Np_52–59_-infected CMT.64 or CMT.TAP1,2/K^b^ cells for ‘arming’. After washing extensively to discard the potential donor cells, the individually armed B-BM-DC populations were injected i.p into separate groups of C57BL/6 mice to elicit VSV-Np antigen immunized carriers. The experiment was repeated twice and the results from a representative experiment are depicted in (Fig. 7A). The results demonstrate that B-BM-DCs armed and loaded with CMT.TAP1,2/K^b^-derived antigens primed an immune response (60% killing) greater than B-BM-DCs loaded with CMT.64-derived antigens (50% killing). These results were similar to *in vitro* ^51^Cr-release assay using C-BM-DCs as immune stimulators (Fig. 7B). In addition to viral infected tumor samples for DC uptake, samples from CMT.64 cells pulsed with different amounts of VSV-Np_52–59_ peptide were also used to evaluate DC-uptake. We performed a dose response of CMT.64 cells pulsed with varied amounts of the peptide in order to examine the threshold sensitivity of CTL recognition and killing. These experiments provided information regarding the amounts of the K^b^/VSV-Np complexes on the cell surface that are required for recognition by antigen specific CTL and allowed us to extrapolate the amounts of MHC-I/peptide complexes that would be available for transfer to DCs. Figure 7C (left panel) shows that killing of CMT.64 cells pulsed with 0.01, 0.5 or 5 µM VSV-Np_52–59_ peptide were epitope-dose dependent, suggesting that the 0.01∼5 µM peptide concentration range titrated and assembled different numbers of K^b^ molecules with peptides on the surface of CMT.64 cells in a dose dependent fashion. Thus, different amounts of K^b^/VSV-Np complexes were available for uptake by DCs. To determine if different amounts of K^b^/VSV-Np complexes taken up by DCs induced T cells with different levels of killing activities, DCs co-cultured with γ-irradiated CMT.64 cells pulsed with 0.01, 0.5 or 5 µM peptide followed by extensive washing were inoculated separately into three groups of mice. The VSV-Np epitope specific T cells were generated by immunization of mice with γ-irradiated 39.5/K^b^ cells pulsed with VSV-Np_52–59_ peptide. Results from Figure 7C (right panel) demonstrate that the three bulk-cultures of VSV-Np-epitope elicited specific T cells exhibited different capacities for killing peptide-pulsed target cells and that killing activity of T cells was dependent on the number of K^b^/VSV-Np complexes from the peptide-pulsed CMT.64 cells available for DC uptake during T cell induction. The greater the number of complexes generated, the more heightened the T cell killing activity. These results suggested that the transfer of surface MHC-I molecules from tumors or infected cells to DCs participated in antigen specific T cell priming *in vivo*.

## Discussion

Priming T cell generation is a key initial step in T cell-based immune responses against tumor cells and pathogens [18], [25]. Here we show that reconstituting expression of TAP and MHC-I in γ-irradiated tumor cells or in virally infected cells can prime and increase the killing activities of antigen specific T cells and can also increase the survival rates of tumor-bearing mice. These findings are highly correlated with cross-presentation of the pathogen or tumor's MHC-I/peptide complex via acquisition, insertion and re-expression into the plasma membrane of newly ‘armed’ BM-DCs. Such observations may provide important information for augmenting the induction phase of adaptive T cell immunity, thereby increasing the efficacy of tumor and pathogen specific immune responses.

The amount of available antigen is an important parameter for induction of adaptive T cell responses [26]. Generally, increasing antigen concentration will prime higher activities of antigen specific T cells. For example, immunization of mice with high, but not low, amounts of chicken ovalbumin protein [27] or apoptotic tumor cells [26] will induce increased T cell generation, and the degree of T cell activation correlates with the dose of antigen acquired by DCs [28]. Bullock et. al also showed that DCs pulsed with higher concentrations of immunogenic peptides enhanced the number of activated CD8^+^ T cells [29], [30]. The results of our experiments demonstrate that the increased activity of T cells is not simply dependent on the amount of antigen expressed but rather the availability of these antigens processed onto the surface of donor tumor cells or infected cells. Expression of TAP and K^b^ molecules allows both VSV-Np_52–59_ and the tumor antigenic peptides to be transported through the secretory pathway for binding to K^b^ molecules, thereby displaying the antigenic peptide/K^b^ complex on the cell surface. Thus, the complexes of surface antigenic peptide-loaded K^b^ molecules provide an available source of preprocessed antigen, that upon transfer to a DC, can enhance T cell priming.

Our results demonstrate that surface MHC-I molecules were directly acquired and taken up by BM-DCs and inserted into their plasma membranes for display to T cells. The phenomenon of uptake of surface MHC-I may involve the transfer of plasma membrane fragments from tumor cells to DCs as shown in Fig 6B (bottom panel). It is still not clear why surface localization of GFP-tagged-K^b^ on the DCs cannot be observed by fluorescence microscopy while it can be detected by cytotoxicity assays (Fig. 6A), but likely relates to relative sensitivity of the analytical procedures. Thus, since the majority of the GFP- tagged-K^b^ tumor cells weekly expressed GFP, it is possible that after DC uptake and display, the relatively small numbers of K^b^ molecules/peptide complexes that were transferred were difficult to observe with fluorescence microscopy techniques. The cytotoxicity assay is a very sensitive method facilitating the detection of the K^b^ molecules [1]. Alternatively, the process and transfer of K^b^ molecules onto the DC surface may be time dependent and this has not been optimized under our present experimental paradigm.

Different levels of MHC-I/peptide complexes produced by DCs pulsed with CMT.64 or CMT.TAP1,2/K^b^ cells can yield varied levels of antigen specific T cells. However, the killing activities of T cell populations are dependent on the levels of MHC-I/peptide complexes taken up by DCs for stimulation of primary T cells as suggested by the results shown in Fig. 7C. These results provide an explanation why immunization with MHC-I proficient (CMT.TAP1,2/K^b^) cells can increase the generation and production of T cells resulting in immune responses greater than those adaptive T cell responses generated by immunization with MHC-I deficient (CMT.64) cells. It is noteworthy and surprising that the different cytotoxic killing levels of effector T cells did not always correlate with the frequency of antigen specific T cell populations (Fig. 5). Such a correlation has often been observed by others [25], [31], [32]. Thus, our results suggest that the MHC-I/peptide complexes taken up and displayed by DCs can activate and ‘tune’ the immune system in a unique or complementary fashion to traditional forms of exogenous and endogenous antigen processing pathways. This process acts to augment the generation of antigen specific T cell subsets with enhanced cytolytic activity.

In the present study, results obtained from peptide-based and protein-based VSV-Np antigens showed differences in T cell activities. Cells infected with high doses of peptide-based antigen, induced higher cytolytic killing activities of antigen specific T cells as well as increased killing activity of T cells in a TAP and MHC-I dependent manner, while this pattern was observed only in protein-based antigens at the lowest dose. Two important questions are raised from these results. First, why are high doses of VSV-Np protein-based antigen incapable of priming a T cell population with higher killing activity? Secondly, why is the increased T cell response in TAP and MHC-I dependent T cell response detected only at the lowest dose of VSV-Np protein-based antigen? Since VSV-Np protein is derived from an infectious pathogen, high levels of VSV-Np (protein-based) but not the (peptide-based) VSV-Np mini-antigen may be directly cytotoxic to components of the immune system. This possibility is supported by a recent report indicating that a novel influenza virus mitochondrial protein can kill host immune cells responding to the influenza viral infection [33]. However, vaccinia virus-derived proteins appear to be nontoxic to the immune system, as the minigene-based VV virus can induce higher killing activities of VSV-Np specific T cells at higher doses of infection. Alternatively, there may be competition for antigen processing at higher doses of infection between the vaccinia viral protein antigens and the VSV-Np protein. This competition would not exist in peptide-based T cell priming because the cells infected with the minigene-based VV can immediately synthesize peptide epitopes for ER-lumen transport and MHC-I binding. The answer to the second question may be explained by DC-based cross-presentation. Antigen uptake by DCs not only includes proteins, peptides, and molecular chaperone-associated peptides [34]–[37] but also surface MHC-I/peptide complexes [present results and [20]]. As discussed above, the latter event explains why TAP and MHC-I expressing tumor cells can prime a better a T cell response in peptide-based T cell priming. Once VSV-Np_52–59_ peptides are synthesized in the cells, only small amounts of the peptide that associate with K^b^ and molecular chaperones [34] can escape the protease-hydrolysis processing and prime T cell generation via the cross-presentation pathway [35]–[37]. Thus, under these conditions, the high number of surface K^b^/peptide complexes becomes an important additional antigen source for T cell priming. In contrast, in cross-presentation of whole protein-based antigens, the large amount of VSV-Np protein taken up by DCs provides a source of antigens that alone may be enough for cross-presentation and T cell priming. Thus, antigen presentation following processing of whole protein-based antigens and the subsequent transfer of MHC-I/peptide complexes to DCs may be less evident.

By using the VSV-Np model antigen, we have demonstrated that DCs efficiently cross-present antigens from TAP and MHC-I expressing donor tumor cells or infected cells for T cell generation. Increased survival of mice immunized with γ-irradiated MHC-I proficient tumor (CMT.TAP1,2/K^b^) cells is likely due to this mechanism. A conflicting report exists from Huang et al [15] who used the MHC-I negative B78H1 and its MHC-I proficient transfectant (derived from B16 melanoma) as a model system to immunize mice and then challenged with MHC-I positive B78H1 cells. They demonstrated that both immunized mouse groups had similar survival rates and confirmed that the immune responses against tumor cells were CD8 positive T cell dependent, as judged by pre-treatment of mice with Ab against CD8 [15]. There are two possible reasons for the discrepancy between these results and our present study. B78H1 cells and CMT.64 cells may express different levels or types of tumor antigens accessing different pathways of antigen processing [38]. If B78H1 cells express high levels of a particular tumor antigen while CMT.64 cells express low levels, the surface MHC-I on relevant MHC-I transfectants may play different roles in DC-based cross-priming. As suggested by our VSV-Np protein-based experiment (Fig. 4), in donor tumor cells expressing whole protein antigen, surface MHC-I/peptide complexes will have a less observable effect on DC cross-presentation. Alternatively, it is likely that B78H1 and its MHC-I transfectant are not only sensitive to CD8 T cells but also to NK and CD4 T cells as well [39]. This was confirmed, in a later study by the same group where they showed that B78H1 cells lacked TAP2 expression [40] become sensitive to NK-mediated killing. As a result, these immune components may provide reinforcement for the antigen specific tumor immune response and thus the effect of surface MHC-I expression on CD8^+^ T cell priming becomes less evident.

In summary, our results demonstrate for the first time that MHC-I/antigenic peptide complexes derived from TAP and MHC-I expressing cells but not TAP and MHC-I deficient cells can enhance T cell priming and subsequently increase the cytolytic activity of antigen specific T cell populations. Operationally, this serves to reduce mortality in tumor bearing mice. The effect of the increased capacity for induction of T cell generation is apparently associated with sampling the MHC-I/peptide complexes expressed on the surface of tumor and virally infected cells that is donated to recipient DCs and then displayed at their cell surface for priming of T cell responses. The newly identified mechanism antigen processing, leading to T cell induction, appears to be distinct from surrogate antigen processing and peptide secretion described previously [41], but appears analogous to trogocytosis [24], and possibly to cross-dressing [42]. This pathway provides additional antigen reservoirs of MHC-I/peptide complexes enabling DC cross-priming. Subversion of this DC ‘arming’ mechanism appears to drive immune-escape and metastasis. Finally, the phenomena we have discovered, likely plays a role in provoking responses to other pathogens and perhaps in autoimmune responses and self-tolerance mechanisms.

## Materials and Methods

### Animals

The C57BL/6 (H-2^b^) and BALB/c (H-2^d^) mouse strains were obtained from NCI and housed in the animal facility at LSU Health Sciences Center. All mice used for the experiments were 6–10 wk old females and were maintained in pathogen-free facilities and treated in accordance with the guidelines of Animal Use Committee at LSU Health Sciences Center.

### Vectors

Vaccinia virus (VV) vectors carrying an entire VSV-nucleocapsid gene (VV-VSV-Np), an epitope-minigene (VV-VSV-Np_52–59_) or a signal sequence-linked epitope-minigene (VV-ss-VSV-Np_52–59_) were kindly provided by Dr. Yewdell [23] (Laboratory of Viral Diseases, National Institute of Allergy and Infectious Diseases, Bethesda, MD). Mouse TAP1 and TAP2 vectors were provided by Dr. Wang (Institute of Cell and Molecular Science, Barts and London School of Medicine, London, UK). Mouse TAP1 and TAP2 cDNAs were isolated and inserted into the vectors pcDNA3.1/His and pUB6/V5-His (Invitrogen Corporation, Carlsbad, CA), respectively. A K^b^-vector was provided by Dr. Flavell (Section of Immunobiology, Yale University School of Medicine, New Haven, CT) and the K^b^ cDNA was isolated and inserted into the vector pEF4/Myc-His (Invitrogen). A GFP-tagged K^b^ vector containing neomycin selected-marker was provided by Dr. Edidin [42] (Department of Biology, Johns Hopkins University, Baltimore, Maryland).

### Cell lines and cell culture

The mouse lung carcinoma cell line CMT.64 was kindly provided by Dr. Gunnel Hallden (Imperial College of Science, London, UK). Four CMT.64 transfectants were generated by transfection with empty vector, mouse TAP1, TAP2 and/or K^b^ gene(s). CMT.TAP1/pEF4 and CMT.TAP1/K^b^ cell lines were generated by co-transfection of TAP1 with either a pEF4/Myc-His empty or a K^b^ vector respectively. Both were maintained in 1000 µg/ml neomycin and 60 µg/ml zeocin selection medium. CMT.TAP1,2/K^b^ and CMT.64/ppp transfectants were generated by co-transfection with TAP1, TAP2 and K^b^ or relevant empty vectors. Both were maintained in 1000 µg/ml neomycin, 60 µg/ml zeocin and 20 µg/ml blasticidin selection medium. A K^b^-transfected D122 lung carcinoma cell line, 39.5/K^b^ [6] was maintained in 500 µg/ml neomycin selection medium. The cell lines were maintained in DMEM medium (Life Technologies, Rockville, MD), while cell lines RMA and RMA-S was cultured in RPMI 1640 medium.

### H-2K^b^ expression

Surface H-2K^b^ expression was detected by a FACScan analyzer (Becton Dickinson, Mountain View, CA) using direct and indirect immunofluorescence. For direct labeling, a PE-conjugated monoclonal antibody (mAb) anti-H-2K^b^ AF6-88.5 and a PE-conjugated anti-H-2K^k^ mAb 36-7-5 (BD Biosciences, San Jose, CA) were used. For indirect labeling, an H-2K^b^ specific Y-3 primary mAb and FITC-conjugated goat anti-mouse IgG secondary antibody (Ab) were used.

### Detection of TAP and VSV-Np expression

Expression of mouse TAP1 and TAP2 proteins were determined by Western blots [14]. For TAP1 expression, blots were incubated in a goat anti-mouse TAP1 polyclonal Ab with 1∶1000 dilution (according to instructions of manufacturer) followed by an HRP-labeled bovine anti-goat Ab (1∶2000 dilution) (both from Santa Cruz Biotechnology, Santa Cruz, CA). For TAP2 expression, blots were incubated in mouse antiserum with 1∶1000 dilution followed by an HRP-labeled goat anti-mouse IgG Ab (1∶3000 dilution) (Santa Cruz Biotechnology). Mouse antiserum against mouse TAP2 protein was created for this study by immunizing BALB/c mice with a peptide sequence (mapping at the C-terminus of mTAP2), DGQDVYAHLVQQRLEA-C with a cysteine at the C-terminus, linked to a keyhole limpet hemocyanin (KLH) carrier protein (Pierce Biotechnology, Rockford, IL). This antiserum can detect mouse TAP2 protein in lysates of RMA but not in TAP2 deficient RMA-S and CMT.64 cells. For VSV-Np detection, a rabbit serum (gift from Dr. Luo, University of Alabama at Birmingham, Birmingham, AL) was used at 1∶5000 dilution followed by an HRP-labeled goat anti-rabbit IgG Ab (Jackson ImmunoResearch Lab. West Grove, PA) at 1∶300,000 dilution. For a loading control, the enzyme glyceraldehyde-3-phosphate dehydrogenase (GAPDH) protein was used as described previously [10], [14].

### Generation of cytolytic T lymphocytes (CTL) and cytotoxicity tests

H-2K^b^-restricted VSV-Np_52–59_ epitope specific CTLs were generated by the following protocols: CMT.64, CMT.TAP1, CMT.TAP/K^b^, CMT.TAP1,2/K^b^ or 39.5/K^b^ cells were infected respectively with VV-VSV-Np, VV-VSV-Np_52–59_ or VV-ss-VSV-Np_52–59_ at 1∶20 multiplicity of infection (m.o.i) for 12–16 hours, and the viral infected cells were subjected to 10,000 rads γ-irradiation. After extensive washing, 1×10^7^ cells were injected i.p into a C57BL/6 mouse for immunization. Immunized splenocytes were obtained 7 to 8 days later and used as CTLs in a standard 4-h ^51^Cr-release assay described previously [10]. In some cases, the CTLs were generated by direct immunization of a mouse with 1×10^7^ plaque formation units (p.f.u) VV-VSV-Np_52–59_ virus with or without γ-irradiation (Fig. 3D). For tumor-antigen CTLs, 1×10^6^ cells/mouse γ-irradiated CMT.64 or CMT.TAP1,2/K^b^ cells were injected i.p. into C57BL/6 mice. Procedures and a standard 4 h ^51^Cr-release assay were described previously [10]. The ^51^Cr-labeled targets are described in each figure legend. For each antigen, two or three experiments were performed and the results represented the mean value (mean±SEM) from two or three experiments.

### Detection of frequencies of VSV-Np_52–59_-specific T cells by a tetramer

Tetramer assays were described previously [25]. Splenocytes from the immunized mice as described above were stimulated *in vitro* with VSV-Np_52–59_ peptides for 5 days. After stimulation, the splenocytes were divided into two aliquots; one was used as CTLs in cytotoxicity tests (Fig. 3A and B) and the other was used to measure the frequency of VSV-Np_52–59_-specific CD8^+^ T cells using double staining [after use of purified anti-mouse CD16/32 antibody (clone 93) to block Fc receptor for 10 min, Biolegend, San Diego, CA ] with an FITC-conjugated anti-mouse CD8a antibody, clone 53–6.7 (Biolegend) and an H-2K^b^/VSV-Np_52–59_ tetramer conjugated to R-phycoerythrin (NIH, MHC Tetramer Core Facility, Atlanta, GA) for 1 hour. After staining, the cells were washed extensively with PBS and used for FACS assay. The gating area was set up to include all cells except erythrocytes and debris. The percentage of tetramer positive cells in the splenocytes and in the CD8^+^ cells was calculated as follow: 1) (number of tetramer positive events from each sample - number of non-specific tetramer events from naïve splenocytes)/total accumulated events in each sample; 2) (number of tetramer positive events from each sample - number of non-specific tetramer events from naïve splenocytes)/CD8^+^ events in each sample. Total accumulated events were 1×10^4^ events for all samples. The mean value (mean±SEM) of the results from two experiments is shown.

### Tumor challenge experiments

C57BL/6 mice were immunized i.p. with 1×10^7^ γ-irradiated CMT.TAP1,2/K^b^, CMT.64/ppp (experimental control) cells, or PBS (negative control). After 20 days, mice were challenged i.p. with 2.5×10^5^ cells CMT.TAP1,2/K^b^ cells and the time of morbidity was recorded. Each group contained 10 mice. Statistics for mouse survival were performed using the Kaplan–Meier log rank survival test and considered different if *p*<0.05.

### Preparation of BM-DCs

BM-DCs were cultured as described previously [43]. BM-DCs were generated from 6 to 8 week old C57BL6 (H-2^b^) and BALB/c (H-2^d^) female mice. On day 6 of *in vitro* culture, immature BM-DCs were exposed for 6 hours with γ-irradiated tumor cells (1∶1 ratio) that were infected with 1∶10 m.o.i VV-VSV-Np_52–59_ overnight and were subsequently subject to maturation with LPS. Mature BM-DCs were used either for T cell generation or as targets for an *in vitro* cytotoxicity assay. For T cell generation, a naïve mouse was inoculated i.p. with 3×10^5^ DCs for 7 days, and splenocytes from the immunized mice were cultured *in vitro* for 4–5 days supplied with VSV-Np_52–59_ peptide and used as effectors. DCs used as targets were labeled with ^51^Cr (as described above). The mean value (mean±SEM) of the results from two experiments is shown.

### *In vivo* cytotoxicity assay

A C57BL/6 mouse was injected with 3×10^5^ antigen-loaded BM-DCs from a BALB/c mouse for immunization (see above for antigen-loading) and after 7 days the mouse was used for *in vivo* cytotoxicity assay.

RBC-lysed, single-cell splenocytes from naïve C57BL/6 mice were pulsed at 1×10^7^ cells/ml with or without 20 µM VSV-Np_52–59_ peptide in DMEM containing 10% FBS for 30 min at 37°C. Afterwards, the cells were washed with PBS twice and then labeled with different concentrations of CFSE [0.5 µM for the cells without peptide-loading (lo), or 5 µM for the cells pulsed with peptide (hi)] at 2×10^7^ cells/ml in PBS for 10 min. CFSE labeling was stopped by addition of three-times volume of PBS containing 2% FBS for 1 min. After washing and re-suspension, 1×10^7^ cells of each population were mixed at a concentration of 4×10^7^cells/ml and 0.5ml was injected i.v. into immunized and non-immunized syngeneic mice. Five hours later, the mice were sacrificed, and the spleens were harvested. Single-cell suspensions of splenocytes were prepared and analyzed by FACS. Percent specific lysis of fluorescent splenocytes in each mouse was calculated as follows: [1−(X_immunized mouse_/X_naïve mouse_)]×100%, where X = hi/lo. Two experiments were performed.

### Determination of uptake of MHC-I from tumor cells by dendritic cells

Uptake of MHC-I from tumor cells by DCs was detected by multi-channel fluorescence microscopy and FACS assay using a GFP-tagged K^b^ system. CMT.64 and CMT.TAP1,2/K^b^ cells were transiently transfected with a GFP-tagged K^b^ vector overnight and were then γ-irradiated (10000 rads). The cells were then frozen at −20°C for one hour and incubated immediately with BM-DCs (DCs were cultured on a cover-slip) at a 2∶1 ratio of tumor cells to DCs. After a 6-hour incubation, DCs were subjected to maturation by adding LPS into the DC culture overnight. The DCs were then pre-incubated with an antibody against CD16/32 (Biolegend) for 10 min to block Fc receptor and labeled with Alexa Fluor 647 anti-mouse CD11c antibody. Uptake of GFP-tagged-K^b^ by DCs was visualized using a Zeiss AxioObserver Z1 widefield microscope. For FACS assay, DCs were labeled with PE-conjugated anti-mouse CD11c antibody (Biolegend). CD11c positive cells were gated as a DC population for detection of uptake of GFP-tagged K^b^. Note: transient, but not stable, transfection of tumor cells with the GFP-tagged K^b^ vector for our experiments was due to the impossibility of generating a stable transfectant from CMT.TAP1,2/K^b^ cells because this cell line already contains a neomycin marker from the TAP1-vector (pcDNA3.1/His vector contains a neomycin marker).
